# Influence of Aging on the Fracture Characteristics of Polyetheretherketone Dental Crowns: A Preliminary Study

**DOI:** 10.3390/polym14194123

**Published:** 2022-10-01

**Authors:** Wen-Ju Lu, Viritpon Srimaneepong, Chiang-Sang Chen, Chang-Hung Huang, Hui-Ching Lin, Chia-Fei Liu, Her-Hsiung Huang

**Affiliations:** 1 Department of Dentistry, National Yang Ming Chiao Tung University, Taipei 112, Taiwan; 2 Department of Prosthodontics, Faculty of Dentistry, Chulalongkorn University, Bangkok 10330, Thailand; 3 Department of Orthopedic Surgery, Far Eastern Memorial Hospital, New Taipei City 220, Taiwan; 4 Department of Materials and Textiles, Asia Eastern University of Science and Technology, New Taipei City 220, Taiwan; 5 Department of Medical Research, MacKay Memorial Hospital, Taipei 251, Taiwan; 6 Department of Stomatology, Taipei City Hospital, Zhongxing Branch, Taipei 103, Taiwan; 7 General Education Center, University of Taipei, Taipei 106, Taiwan; 8 Institute of Oral Biology, National Yang Ming Chiao Tung University, Taipei 112, Taiwan; 9 Department of Medical Research, China Medical University Hospital, China Medical University, Taichung 404, Taiwan; 10 Department of Bioinformatics and Medical Engineering, Asia University, Taichung 413, Taiwan; 11 Department of Education and Research, Taipei City Hospital, Taipei 103, Taiwan; 12 Department of Stomatology, Taipei Veterans General Hospital, Taipei 112, Taiwan

**Keywords:** polyetheretherketone, TiO_2_, aging, dental crown, fracture

## Abstract

Although polyetheretherketone (PEEK) is becoming more widely used in dentistry applications, little is known about how aging will affect this material. Therefore, this study aimed to investigate the influence of an aging treatment on fracture characteristics of PEEK dental crowns. Additionally, the impact of the addition of titanium dioxide (TiO_2_) into PEEK was examined. Two types of commercial PEEK discs were used in this study, including TiO_2_-free and 20% TiO_2_-containing PEEK. The PEEK dental crowns were fabricated and aging-treated at 134 °C and 0.2 MPa for 5 h in accordance with the ISO 13356 specification before being cemented on artificial tooth abutments. The fracture loads of all crown samples were measured under compression tests. Results demonstrated that adding TiO_2_ enhanced the fracture load of PEEK crowns compared to TiO_2_-free PEEK crowns before the aging treatment. However, the aging treatment decreased the fracture load of TiO_2_-containing PEEK crowns while increasing the fracture load of TiO_2_-free PEEK crowns. The fracture morphology of TiO_2_-containing PEEK crowns revealed finer feather shapes than that of the TiO_2_-free PEEK crowns. We concluded that adding TiO_2_ increased the fracture load of PEEK crowns without aging treatment. Still, the aging treatment influenced the fracture load and microscopic fracture morphology of PEEK crowns, depending on the addition of TiO_2_.

## 1. Introduction

Polyaryletherketone (PAEK) is a high-performance thermoplastic polymer family which contains aromatic hydrocarbons and a different proportion of ketones and ether groups [1]. In addition to having exceptional dimensional stability, superior wear resistance at high temperatures, and less wear of the antagonist’s teeth, they also have strong tensile, fatigue, and bending strengths with good color stability [2]. Moreover, the radiolucency of this thermoplastic polymer means that they do not induce artifacts in medical imaging [3]. Polyetheretherketone (PEEK) is one of the most commonly used polymers in the PAEK family in medicine and mainly serves as a substitute for metal orthopedic implants [4]. In recent years, PEEK has been applied to dental applications in oral implantology, such as implant abutment, and dental prostheses, such as crowns or partial dentures [5,6,7].

However, PEEK has a relatively lower compressive strength than cortical and alveolar bone; thus, previous studies experimented with adding different fillers, such as TiO_2,_ into the PEEK matrix to improve its mechanical properties [8,9,10]. In addition, TiO_2_ particles also serve as white pigment to adjust the color of PEEK. Previous research has shown that adding TiO_2_ particles to PEEK would improve its hardness performance [8] and increase the bending coefficient of PEEK simultaneously [9]. Furthermore, one study showed that PEEK containing 20% TiO_2_ particles has a significantly higher compressive strength than PEEK with 10% TiO_2_ particles [10]. To enhance the mechanical properties of PEEK, different inorganic particles, such as Al_2_O_3_, Cu_2_O, AlN, and Si_3_N_4_, modified phosphates are also added to the PEEK matrix [11].

One recent study on the mechanical properties of PEEK focused on static mechanical tests, while dental prostheses were subjected to prolonged humidity and temperature change in oral cavities [12]. The aging of the prostheses over a long period of time in the warm and moist environment in the mouth should be considered during the design process. There are many studies on the effect of aging treatments on the various properties of dental materials, either dental composites or zirconia [13,14,15], but there is no evidence of an aging effect on the properties of PEEK. It is important to consider that aging significantly impacts the mechanical properties of PEEK dental prosthetics. Therefore, the objective of this study was to investigate the influence of aging on the fracture characteristics of PEEK dental crowns with and without the addition of TiO_2_ particles.

## 2. Materials and Methods

### 2.1. Sample Preparation of PEEK Dental Crown

Two kinds of commercially available PEEK discs for dental application were investigated, including PEEK without the addition of the TiO_2_ particles (Schütz Dental, Rosbach vor der Höhe, Germany) and PEEK with 20% TiO_2_ particles (Denracle, Tainan, Taiwan). The composition and some basic mechanical properties provided by the manufacturers were listed below: PEEK without TiO_2_ containing 100% PEEK with elastic modulus > 4.0 GPa and tensile strength > 100 MPa; PEEK with TiO_2_ containing 80% PEEK+ 20% TiO_2_ with elastic modulus~4.6 GPa and tensile strength~97 MPa.

**Figure 1:**
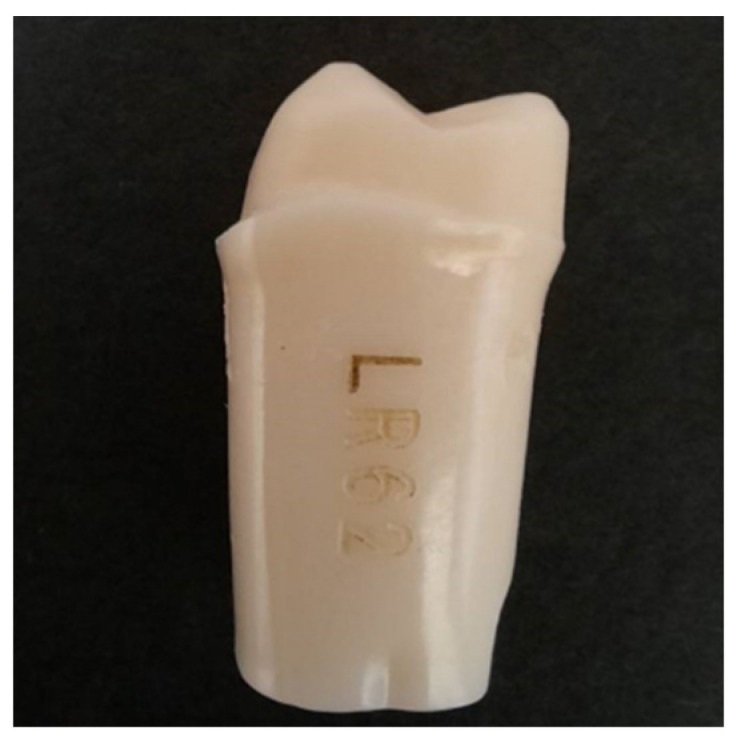
Artificial tooth abutment (LR 62, Nissin Dental Products, Inc., Kyoto, Japan) used in this study.

The abutment tooth for the PEEK crown was prepared on the artificial lower right mandibular first molar (#46) (LR 62, Nissin Dental Product, Kyoto, Japan) (Figure 1) with the finishing line (margin) of the tooth preparation thickness of 0.50 ± 0.05 mm and occlusal surface thickness 1.0 ± 0.1 mm following the recommended tooth preparation for the zirconia crown. Then, each artificial tooth abutment was directly scanned using a scanner (inEos X5, Dentsply Sirona, Charlotte, NC, USA), and a computer-aided design (CAD) system (built-in software, 3Shape, København, Denmark) was used to create the consistent shape of the crown. All PEEK crowns were produced by computer-aided manufacturing (CAM) system (MC X5, Dentsply Sirona, Charlotte, NC, USA).

Therefore, all prepared artificial tooth abutments matching the corresponding milled crowns were identical. The PEEK crown samples from each manufacturer were divided into two groups: (1) the control group did not undergo the aging treatment, and (2) the aging treatment group was autoclaved at 134 °C and 0.2 MPa treatment for 5 h to simulate the long-term moist oral environment in accordance with ISO 13356.

### 2.2. Fracture Load Measurement of PEEK Crown

**Figure 2:**
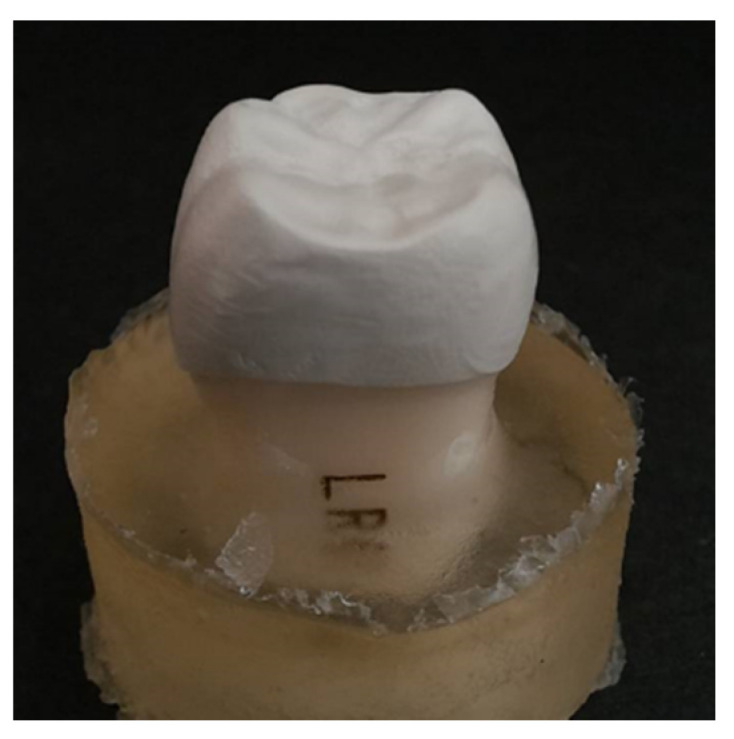
PEEK dental crown was cemented on artificial tooth abutment and embedded in epoxy for compression test.

**Figure 3:**
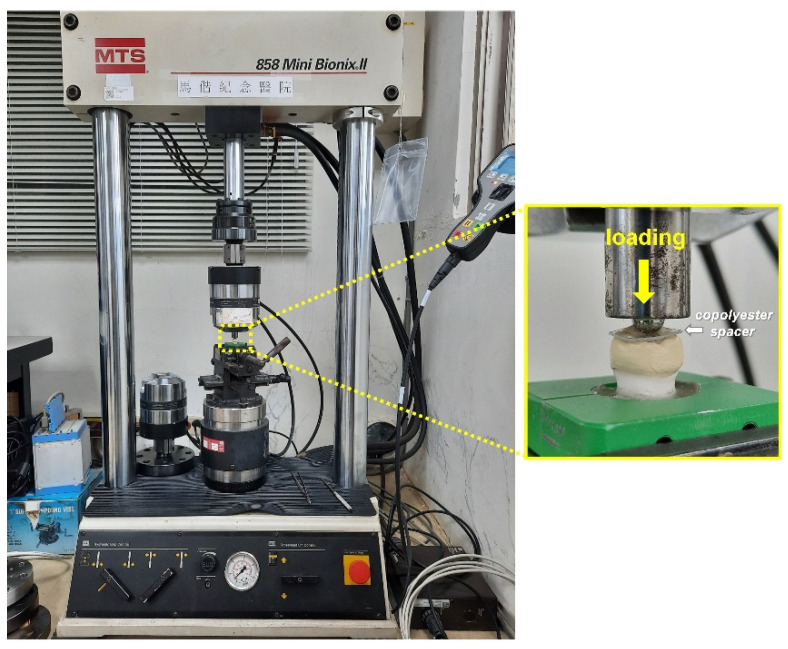
Compression test machine (**left**) and higher magnification of test PEEK crown during loading (**right**).

Before bonding on artificial tooth abutments, the dimensions at the reference points of each CAD/CAM-produced PEEK crown were measured and confirmed. All PEEK crown samples were cemented on the artificial tooth abutments using a medical grade epoxy adhesive (EA M-31 CL, Loctite, Rocky Hill, CT, USA) instead of a clinical resin luting cement to gain maximum bonding between PEEK crown and artificial tooth abutment. Even though this epoxy adhesive passed the ISO 10993 biological tests [16] and an in vivo study used this adhesive for sealing dental post space [17], this medical-grade adhesive in this in vitro study was not intended for clinical implication. The abutments with cemented PEEK crowns were embedded in epoxy (EpoFix Kit, Struers, Rødovre, Denmark) for the compression test (Figure 2). The compression test was carried out using a universal tester (Bionix-858, MTS, Eden Prairie, MN, USA) with a loading crosshead speed of 1 mm/min to determine the fracture loads of the crown samples (Figure 3). The 0.5 mm thick copolyester spacer (Imprelon^®^ Spd, Scheu-Dental GmbH, Iserlohn, Germany) was used between the loading head and occlusal surface of the PEEK crown to distribute stress evenly on the occlusal surface of the PEEK crown during a compressive test. The fracture load was measured upon the cracking of samples occurred. The number of test samples for each group per compression test was three. Each compression test was performed in duplicate. The fracture load data is based on the results of two repeated compression tests. The fracture load data was based on the results of two repeated compression tests. However, we did not perform precise statistical analysis based on the limited sample size.

### 2.3. Analyses of Chemical Element in PEEK Matrix and Fracture Morphology of PEEK Crown

We used an energy dispersive spectrometer (EDS) (Ultim Max 100, Oxford Instruments, Abingdon-on-Thames, UK) to conduct a semi-quantitative analysis of carbon (C), oxygen (O), and titanium (Ti) elements in the PEEK matrix after surface polishing to confirm the presence and distribution of the TiO_2_ particles for each group. After compression tests, the macroscopic fracture morphology of the PEEK crowns was first visually observed. To assess the fracture characteristics, the fracture surfaces were coated with a 25 nm thick conductive platinum film, and the microscopic fracture morphology was observed using scanning electron microscopy (SEM) (JSM-6500F, JEOL, Tokyo, Japan).

## 3. Results and Discussion

### 3.1. Analysis of Chemical Elements in PEEK

**Figure 4:**
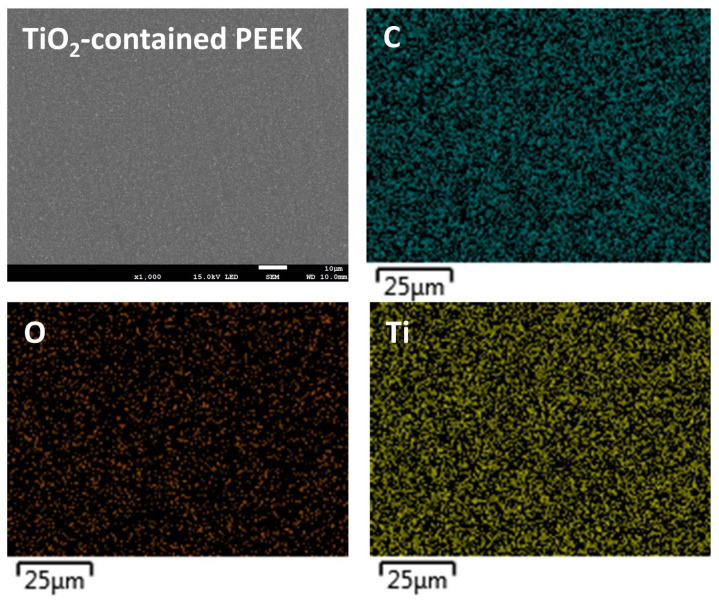
SEM micrograph of surface morphology of 20% TiO_2_-containing PEEK (upper-left) and the corresponding EDS mapping analysis of the C, O, and Ti elements.

Figure 4 presents the SEM micrographs of the 20% TiO_2_-containing PEEK polished surface and the corresponding EDS mapping analysis of the C, O, and Ti elements. The bright color spots indicated relatively higher element contents. The findings of the EDS analysis revealed that the Ti and O elements were dispersed evenly in the TiO_2_-containing PEEK matrix. This confirmed that the TiO_2_ particles were distributed evenly throughout the PEEK matrix.

### 3.2. Effect of Aging Treatment on Fracture Load of PEEK Crowns

**Figure 5:**
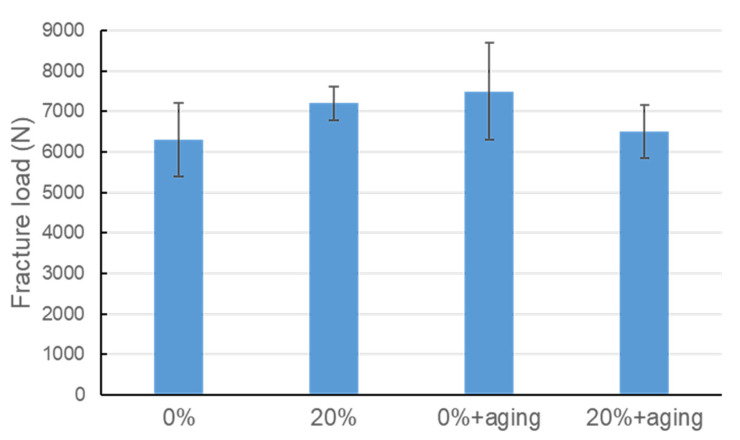
Fracture loads of test PEEK crowns after compression tests, including 0% TiO_2_ (without aging), 20% TiO_2_ (without aging), 0% TiO_2_+aging, and 20% TiO_2_+aging.

Figure 5 presents the fracture loads of test PEEK crowns with and without aging treatment. The results demonstrated that the 20% TiO_2_-containing PEEK crowns had a higher fracture load (~7200 N) than the TiO_2_-free PEEK crowns (~6300 N) before the aging treatment. This finding could be related to the even distribution of the TiO_2_ particles in the PEEK matrix and thus increased the mechanical properties. This would lead to better load absorption and higher fracture resistance. Similar previous results indicated the mechanical properties of PEEK improved with the inclusion of the TiO_2_ nanoparticles [8,9,10]. They also suggested that as the content of the TiO_2_ increases, the bending coefficient and hardness of the PEEK bar samples increase [8,9], but the compressive strength of cylindrical PEEK samples also improved [10].

One report showed that the PEEK crowns subjected to cyclic loading did not significantly alter their fracture loads [18]. However, there is no detailed information on how aging affects the mechanical properties of PEEK in the literature. In this investigation, we found that the fracture load of the TiO_2_-containing PEEK decreased after the aging treatment, in contrast to the TiO_2_-free PEEK group (Figure 5). This may imply that the aging treatment had an adverse effect on the fracture load of PEEK, depending on the presence of TiO_2_. The aging treatment decreased the fracture load of 20% TiO_2_-containing PEEK crown from approximately 7200 N to 6500 N.

In contrast, the fracture load of the TiO_2_-free PEEK crowns increased from approximately 6300 N to 7500 N after aging treatment. We speculated that the presence of the TiO_2_ particles in the PEEK matrix may negatively affect the mechanical properties during the aging treatment. However, the underlying mechanism still needs to be further investigated. Note that the aging treatment specified in ISO 13356 is expected to simulate an approximately 10-year moist oral environment [19].

In this study, the fracture loads of PEEK crowns in all groups ranged from approximately 6300–7500 N, which were far greater than the average human bite force (about 300 N) [20] and were also higher than those (3000–6000 N) obtained in a study by Shirasaki et al. [18]. It was approximately a 300–4500 N difference in fracture load between the data from this study (Figure 5) and the previous study by Shirasaki’s group [18]. The difference in fracture load could be due to the different testing preparations, abutment scanning methods, and PEEK materials. We also speculated that the cementation and abutment materials selected for the fabrication of PEEK crown samples may also have influenced the results, even though the PEEK crown samples for compression tests were prepared based on the clinical manufacturing procedure. 

Our findings could be explained by the possibility that TiO_2_ nanoparticles in the PEEK matrix may act as a load absorber, leading to an increase in fracture load. However, these nanoparticles would have a detrimental effect when subjected to high temperature and pressure during the aging treatment. High temperatures and moisture could have a negative impact on the bond between the TiO_2_ nanoparticles and PEEK matrix—further investigation on the mechanism behind this is needed. Therefore, our results could echo the recent systemic review that there is insufficient data to support PEEK dental prostheses’ long-term survival [21].

Considering the current clinical trend in using zirconia crowns, we applied the same methodology to a zirconia crown (#46) to measure the corresponding fracture load by a compression test [22]. The results showed that the PEEK crowns obtained in this study (without the aging treatment) had two to three times the fracture load of the zirconia crowns.

### 3.3. Macroscopic Observations of Fracture Morphology of PEEK Crowns

**Figure 6:**
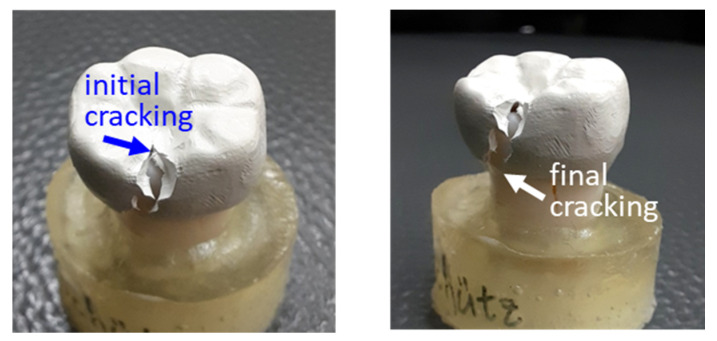
Visually macroscopic observation of the fracture morphology of PEEK crown after compression test: initial cracking (**left**) and final cracking (**right**).

The visual, macroscopic observation of fracture morphology of the TiO_2_-free PEEK crown after the compression test is shown in Figure 6. Visual observation revealed that the fracture of the PEEK crown started from the lingual side of the occlusal surface and extended from one-third of the lingual groove to the lingual side of the crown. It can be inferred that the weakest part of the entire PEEK crown structure of the lower jaw’s first molar is the lingual groove. A similar visual fracture morphology was also seen in the PEEK crowns containing 20% TiO_2_. This is because the weakest part of the crown is where the crown is the thinnest, which must be considered during crown design and preparation.

### 3.4. Microscopic Observations of Fracture Morphology of PEEK Crowns

**Figure 7:**
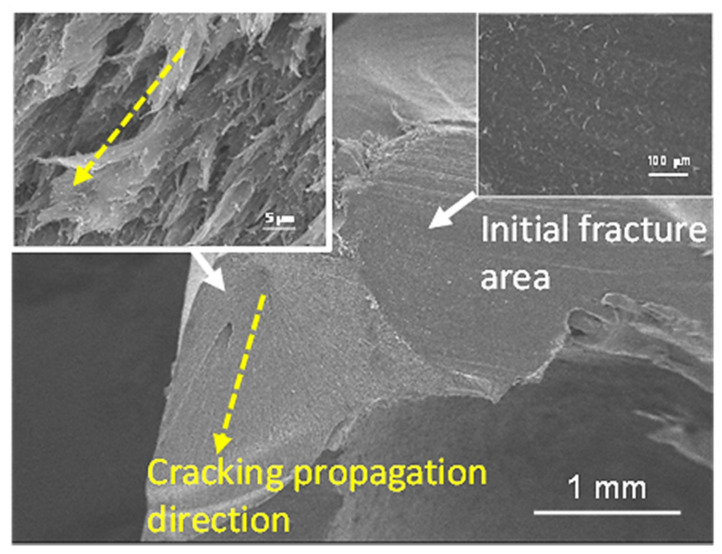
SEM microscopic observation of fracture morphology of the TiO_2_-free PEEK crown after compression test (yellow arrow indicates the direction of cracking propagation).

**Figure 8:**
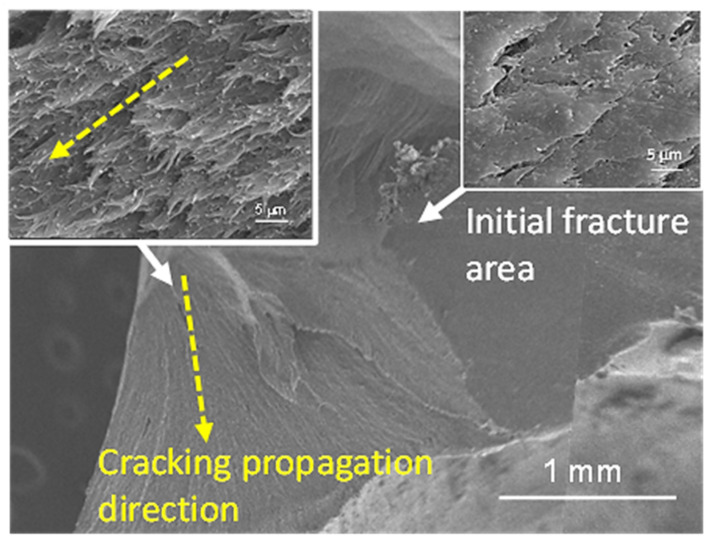
SEM microscopic observation of fracture morphology of 20% TiO_2_-containing PEEK crown after compression test (yellow arrow indicates the direction of cracking propagation).

The SEM microscopic observation of fracture morphology of 0% and 20% TiO_2_-containing PEEK crowns after compression tests are shown in Figure 7 and Figure 8, respectively. Regardless of the presence of TiO_2_, it demonstrated that the fracture surface where the fracture started was relatively smooth (upper-right inset in Figure 7 and Figure 8), followed by the ductile and feather-shaped fracture propagation surface. The feather-shaped morphology of the fracture surfaces of PEEK containing 20% TiO_2_ (upper left in Figure 8) appeared finer (more meticulous) compared to that of the TiO_2_-free PEEK (upper left in Figure 7). According to previous research, the cracked region in the middle fracture portion of the TiO_2_-containing PEEK shows a feather-shaped morphology after tensile tests, as described below [23]. Cracking propagation follows the weaker amorphous structures between the crystalline spherulites in the PEEK matrix, thereby leading to the feather-shaped fracture morphology. The addition of more TiO_2_ particles to PEEK results in smaller spherulites and finer amorphous structures; therefore, the feather-shaped fracture morphology is more meticulous.

## 4. Conclusions

Based on the limitation of our preliminary study, the following conclusions are:

1. When aging treatment was not applied, the addition of 20% TiO_2_ particles in the PEEK crown increased the fracture load under the compression test compared to the TiO_2_-free PEEK crown. However, the aging treatment had a different impact on the fracture load of the PEEK crown, depending on the presence of TiO_2_. The aging treatment decreased the fracture load of 20% TiO_2_-containing PEEK crown, whereas the fracture load of the TiO_2_-free PEEK crown increased after aging treatment. The underlying mechanism will be further investigated.
2. The fracture of all test PEEK crowns started from one-third of the lingual groove and extended to the lingual side of the crown. The beginning of the fracture surface was relatively smooth, while the surface in the middle portion of the fracture propagation area was ductile and feather-shaped, particularly more distinct, and of a finer feather shape on the 20% TiO_2_-containing PEEK crown.

## Data Availability

The data presented in this study are available on request from the corresponding author upon reasonable request.
